# Wheat supplement with buckwheat affect gut microbiome composition and circulate short-chain fatty acids

**DOI:** 10.3389/fnut.2022.952738

**Published:** 2022-09-06

**Authors:** Di Yao, Qiaoru Yu, Lei Xu, Tingting Su, Lixue Ma, Xiaoyu Wang, Mengna Wu, Zhijiang Li, Dongjie Zhang, Changyuan Wang

**Affiliations:** ^1^College of Food, Heilongjiang Bayi Agricultural University, Daqing, China; ^2^Heilongjiang Engineering Research Center for Coarse Cereals Processing and Quality Safety, Daqing, China; ^3^Key Laboratory of Agro-Products Processing and Quality Safety of Heilongjiang Province, Daqing, China; ^4^National Coarse Cereals Engineering Research Center, Heilongjiang Bayi Agricultural University, Daqing, China

**Keywords:** buckwheat, cereals mixture, gut microbiome, short-chain fatty acids, *in vitro* fermentation

## Abstract

Buckwheat has beneficial effects on human intestinal health, which is often compounded with wheat to make food. Therefore, the effect of cereals mixture via *in vitro* fermentation on gut microbes and short-chain fatty acids (SCFAs) were investigated in this study. The mixture of wheat and tartary buckwheat (WT) produced more lactate and acetate, and the mixture of wheat and sweet buckwheat (WE) produced more propionate and butyrate. Compared with wheat (WA), the relative abundance of some beneficial bacteria significantly increased, such as *Sutterella* in WT and *Faecalibacterium* in WE. Cereals mixture also affected the expression of functional genes, involved in metabolic pathways and carbohydrate-active enzymes (CAZymes) that modulated SCFAs generation. This study provides new insights into the effects of sweet and tartary buckwheat on intestinal function, which is beneficial to applying both types of buckwheat in practical.

## Introduction

Sweet buckwheat (*Fagopyrum esculentum* L.) and tartary buckwheat (*Fagopyrum tataricum* L.) are main cultivated varieties, which belong to the Polygonaceae (1). Buckwheat is not only rich in protein and starch, but also contains various nutrients, such as phenolic acids, fagopyrins, and other antioxidants (2– 4). Tartary buckwheat has high medicinal value and many beneficial effects on the human body, and cures diseases of the intestines. Tartary buckwheat is rich in rutin and bittern, which have hypoglycemic and hypolipidemic effects (5, 6). During the early stage of sweet buckwheat growth, active accumulation of glutamic acid was shown. It may be related with arginine, proline and leucine accumulation (7). Because sweet buckwheat is rich in amino acids, it has certain benefits for the intestinal flora (8). The dietary fiber contained in sweet and tartary buckwheat can improve diversity of microbial community, promote the growth of SCFAs-producing bacteria in the intestinal tract, maintain the integrity of microbial community structure. Although sweet and tartary buckwheat have good utilization value, they cannot replace the staple cereals owing to rough taste and poor palatability (9). Normally, sweet and tartary buckwheat are often mixed with staple grains to make bread or noodles. Compared to whole wheat, sweet and tartary buckwheat can promote the growth of probiotics and inhibit the number of intestinal pathogenic bacteria (10). Consequently, it is meaningful to study the role of cereals mixture.

*In vitro* fermentation is an effective way to explore intestinal function. *In vitro* fermentation models offer unique advantages, including closely mimicking the microbial composition and activity in the gastrointestinal tract, and have been proposed as alternatives to *in vivo* studies. *In vitro* models are relatively simple, have no ethical constraints, can be effectively controlled, avoid interference by other components and allow for dynamic sampling over time (11). Previous study used to explore the effects coarse grains on gut microbiota by adult fermentation models *in vitro* (12, 13), which is an effective tool for evaluating the impact of prebiotics on gut microbiota (14, 15).

The intestinal flora and its metabolites can play an important role in host immune homeostasis by promoting the development of the immune system, activating the immune response and regulating immune cell function (16, 17). SCFAs are metabolites produced by microbial fermentation in the gut (18). SCFAs are a part of saturated fatty acids containing six or fewer carbon atoms that mainly include acetate, propionate and butyrate (19). SCFAs have a variety of beneficial biological functions, such as maintaining intestinal homeostasis, a low-inflammatory state, and good intestinal barrier function. Traditional 16S rRNA gene-based amplification methods cannot fully understand the composition of the entire microbial community. However, the development of metagenomics has made it possible to study the component, functional genes of human gut microbes and carbohydrate metabolism pathways. Although numerous studies have reported the properties of sweet and tartary buckwheat affecting gut microbes and regulating the production of metabolites such as SCFAs. However, the *in vitro* fermentation of combined buckwheat and wheat has not been reported to regulate the production of SCFAs by intestinal flora and affect functional genes of human gut microbes.

Therefore, this study aimed to reveal the changes in bacterial, fungal and viral community composition, effects of SCFAs on intestinal health and expression of functional gene in metabolic pathways after cereals mixture fermentation *in vitro*. This study provides new ideas for studying the changes in gut microbes and functional genes during *in vitro* fermentation of the cereals mixture, thus being beneficial for the application of the two buckwheat in food.

## Materials and methods

### Source and *in vitro* digestion of cereals mixture

Wheat, tartary and sweet buckwheat were purchased from online stores (Jining, Shandong Province, China). The samples were pulverized by mill (DJJFT-50A, Changsha, China). Tartary and sweet buckwheat were, respectively, mixed with wheat on a ratio of 2:3, named WT and WE. The wheat group was named WA. A 20 g sample was mixed in 180 mL of distilled water, heated and continuously stirred for 20 minutes into a mature state, and cooled to room temperature, then 10 mL of 1,850 U/mL α-amylase was added, and shaked for 20 min in an Orbital Shaking Incubator at 37°C to simulate oral digestion. Adjusted the pH to 2 with 6 mol/L HCl, adding 10 mL of 4,000 U/mL pepsin in 0.1 mol/L HCl, shaked for 30 min at 37°C. Finally, 10 mL of the pancreatin-bile mixture (7,000 U/mL) was added, and pH was adjusted to 7 with 1 mol/L NaHCO_3_. The samples were shaken at 37°C for 6 h. Afterward, the test tubes were immediately immersed in ice for 15 min to stop the enzymatic reaction. Next, the samples were centrifuged to obtain undigested residues. Finally, the physicochemical properties of the samples before and after digestion were examined. The protein (GB5009.5-2016), ash (GB5009.4-2010), starch (GB5009.9-2016), dietary fiber (GB5009.88-2014) and fat (GB5009.6-2016) contents of the samples were determined using national standard methods.

### *In vitro* fecal fermentation

Six healthy donors (age 20–25) with normal BMI (18.5 kg/m^2^ < BMI < 24 kg/m^2^) were recruited in Heilongjiang Bayi Agricultural University, which provided fresh feces samples on the day of the fermentation experiment. Donors had no gastrointestinal disease or were treated with antibiotics for at least 3 months, and signed the informed consent. Feces were collected by feces collector, stored at 4°C, and within 2 h in an insulated bag. The feces were transfered to sterile tubes, and immediately placed inside an anaerobic chamber (80% N_2_, 16% CO_2_ and 4% H_2_). All further procedures were performed within 4 h after collection. The fecal slurry was prepared by mixing fecal samples with carbonate-phosphate buffer at a ratio of 30%, and further filtered through 4 layers of cheesecloth. A fecal suspension was prepared with 0.5 g of the undigested cereals residues and 2 mL of fecal inoculum into test tubes, then 9.5 mL of fermentation medium (1% peptone, 0.025% cysteine, and sodium sulfide) (20) was added to each tube, the expected fecal concentration is 5% (w/v). Another group of fecal suspensions without cereals mixture was set as control and named FS. The *in vitro* fermentation was maintained under anaerobic conditions with orbital shaking (125 r/min) at 37°C for 24 h. Subsequently, the test tubes were soaked in ice for 10 min, and aliquots were taken and stored at −80°C until further processing.

### Determination of short-chain fatty acids

SCFAs were determined by HPLC method. Taking 10 mL of the fecal fermentation liquid in a centrifuge tube, centrifuge at 5,000 r/min for 10 min; taking 5 mL of the supernatant, add 1 mL of 25% (v/v) metaphosphoric acid solution, mixed well and placed in the refrigerator (−20°C) frozen overnight; after the protein in the samples were solidified by metaphosphoric acid acidification, and taking it out and completely thawed it, and the samples were centrifuged at 12,000 r/min for 10 min; the supernatant was filtered through a 0.22 μm water-phase filter and used on the HPLC (Agilent 1200, Agilent Technologies Co., Ltd., America). The determination was performed using a Carbomix H-NP column (5 μm, 7.8*300 mm, Sepax Technologies Co., Ltd., America). The column temperature was 60°C, and the flow rate was 0.5 mL/min. The mobile phase was H_2_SO_4_ + 2.5% (v/v) acetonitrile. The content of SCFAs was qualitatively determined by the retention time of the standard substance, and the peak area was quantified by the external standard method.

### DNA extraction, metagenomic sequencing and genome assembly

Total genomic DNA was extracted from feces samples using the E.Z.N.A.^®^ Soil DNA Kit (Omega Bio-Tek, Norcross, GA, United States) according to manufacturer’s instructions. Concentration and purity of extracted DNA was determined with TBS-380 and NanoDrop2000, respectively. DNA extract quality was checked on 1% agarose gel. DNA extract was fragmented to an average size of about 400 bp using Covaris M220 (Gene Company Limited, China) for paired-end library construction. Paired-end library was constructed using NEXTflexTM Rapid DNA-Seq (Bioo Scientific, Austin, TX, United States). Adapters containing the full complement of sequencing primer hybridization sites were ligated to the blunt-end of fragments. Paired-end sequencing was performed on Illumina NovaSeq/Hiseq Xten (Illumina Inc., San Diego, CA, United States) at Majorbio Bio-Pharm Technology Co., Ltd. (Shanghai, China) using NovaSeq Reagent Kits/HiSeq X Reagent Kits according to the manufacturer’s instructions^1^. Sequence data associated with this project have been deposited in the NCBI Short Read Archive database (Accession Number: SRP372133). The raw reads from metagenome sequencing were used to generate clean reads by removing adaptor sequences, trimming and removing low-quality reads using the fastp^2^ (version 0.20.0) on the free online platform of Majorbio Cloud Platform^3^.

### Species and function annotations

Representative sequences of non-redundant gene catalog were annotated based on the NCBI NR database using blastp as implemented in DIAMOND v0.9.19 with e-value cutoff of 1e^–5^ using Diamond^4^ (version 0.8.35) for taxonomic annotations. The Kyoto Encyclopedia of Genes and Genomes and Carbohydrate enzymes database annotation was contrasted the KEGG^5^ (version 94.2) and CAZymes^6^ (version v8) with an e-value cutoff of 1e^–5^.

### Statistical analysis

For all the analyses, the data were analyzed statistically using the SPSS statistical package version (SPSS Inc., Chicago, IL) and Origin 2021 (Origin Lab Corporation, United States). Analysis of variance (ANOVA, Duncan’s method at a significance level of *p* < 0.05) was applied to the experimental data. For analysis software, the online platform of Majorbio ISanger Cloud platform^7^ was used. The experiment was performed in triplicate and the results were expressed as the mean ± SD.

## Results and discussion

### Analysis of physical and chemical properties

Several physical and chemical properties of cereals mixture before and after digestion were measured (Tables 1, 2). The content of starch after *in vitro* digestion was 30-33 g/100 g (including resistant starch). The digestibility of starch was the highest, was 43-46%. The protein content was 7-10 g/100 g, the digestibility of protein among the three groups was very different, ranging from 26 to 57%, with the highest digestibility of WA and the lowest digestibility of WE. Fat content was 4-8 g/100 g. Fat digestibility was 30-59%. Dietary fiber generally increased after digestion in each group, especially the insoluble dietary fiber of WT (19.98 g/100 g). Dietary fiber was more difficult to digest than starch, protein and fat. Dietary fiber is a plant polysaccharide that cannot be digested by human digestive enzymes, which can play a crucial role in metabolic activity, and can affect gut health and the immune system, as well as the body’s ability to fight certain chronic diseases (21).

**TABLE 1:** Changes of dietary fiber content in each group before and after *in vitro* digestion (g/100 g).

Samples		Dietary fiber		
		Soluble	Insoluble	Total
WA	Before	0.087 ± 0.00^c^	0.154 ± 0.01^b^	0.241 ± 0.04^c^
	After	1.11 ± 0.03^b^	1.05 ± 0.07^c^	0.26 ± 0.05^c^
WT	Before	2.34 ± 0.05^a^	4.43 ± 0.06^a^	6.77 ± 0.05^a^
	After	4.65 ± 0.03^a^	19.98 ± 0.40^a^	24.6 ± 0.40^a^
WE	Before	0.431 ± 0.04^b^	0.228 ± 0.00^b^	0.660 ± 0.01^b^
	After	0.970 ± 0.05^c^	3.653 ± 0.03^b^	4.62 ± 0.03^b^

Letters indicate the significance of differences among groups.

**TABLE 2:** Changes of starch, protein, fat and ash content in each group before and after *in vitro* digestion (g/100 g).

Samples		Starch	DR	Protein	DR	Fat	DR	Ash
WA	Before	58.53 ± 0.39^a^	45.36%	17.35 ± 1.05^a^	56.02%	10.50 ± 0.42^b^	30.76%	1.04 ± 0.11^a^
	After	31.98 ± 0.49^a^		7.63 ± 0.23^c^		7.27 ± 0.08^a^		12.95 ± 0.69^a^
WT	Before	55.57 ± 0.57^b^	43.30%	15.15 ± 0.12^b^	41.45%	9.60 ± 0.31^c^	58.33%	1.14 ± 0.05^a^
	After	31.51 ± 1.19^a^		8.87 ± 0.06^b^		4.00 ± 0.90^b^		13.78 ± 1.42^a^
WE	Before	59.21 ± 1.00^a^	45.40%	13.24 ± 0.88^c^	26.96%	11.79 ± 0.26^a^	36.05%	0.79 ± 0.02^b^
	After	32.33 ± 0.31^a^		9.67 ± 0.15^a^		7.54 ± 0.31^a^		12.12 ± 0.19^a^

Letters indicate the significance of differences among groups.

### Effect of cereals mixture on microbiota composition

**FIGURE 1:**
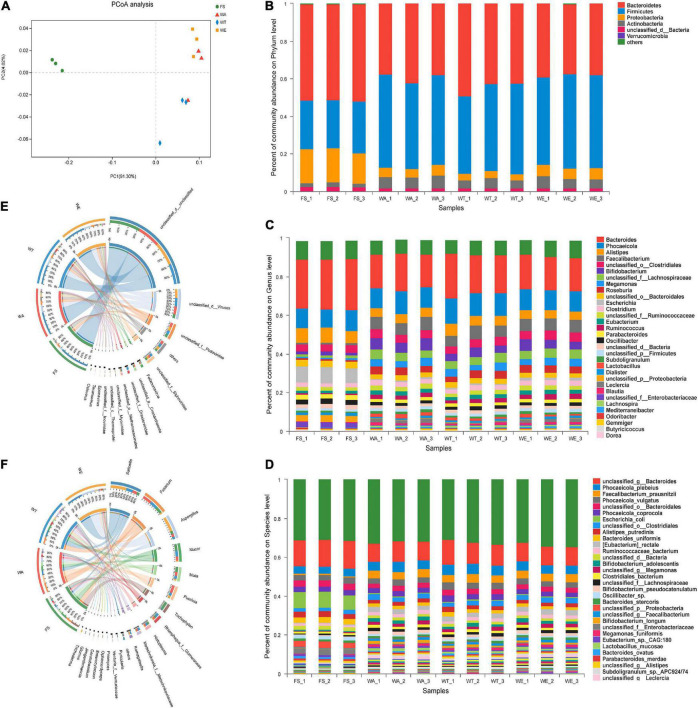
The microbial community distribution and relative abundance of bacteria, fungi and viruses in the cereals mixture and the community differences among groups. **(A)** Principal coordinates analysis (PCoA) of bacteria, (genus level). Some classes with less than 1% abundance were summarized as “Other.” **(B)** Distribution and relative abundance of bacterial communities at the phylum level. **(C)** Distribution and relative abundance of bacterial communities at the genus level. **(D)** Distribution and relative abundance of bacterial communities at the species level. **(E,F)** Distribution and relative abundance of fungi and viruses at the genus level, respectively.

To elucidate the effect of cereals mixture on the microbial flora, after the intervention of different cereals mixture, the alpha-diversity of the four groups of FS, WA, WT, and WE were determined (Table 3). The Ace and Shannon values of bacteria were greater than fungi and viruses in the samples (22). There were differences in Shannon index among the three intervention groups (*P* < 0.05), thus differences in species diversity among the three intervention groups. In addition, the microbial community distribution at the genus level was analyzed by the beta diversity analysis (PCoA). There were significant differences in microbial community structure between FS and the three intervention groups (Figure 1A). The distribution of bacteria in WT and WE were obviously different, but they were partially similar to WA’s community distribution, which may be because WT and WE contain wheat.

**TABLE 3:** Alpha diversity analysis of microbial communities.

Sample	ACE			Shannon			Simpson		
	Bacteria	Fungi	Viruses	Bacteria	Fungi	Viruses	Bacteria	Fungi	Viruses
FS	5580.67 ± 79.66^bA^	19.67 ± 1.53^cB^	138.33 ± 5.86^cB^	4.49 ± 0.02^cA^	2.49 ± 0.13^aC^	3.42 ± 0.05^aB^	0.04 ± 0.00^aB^	0.11 ± 0.02^aA^	0.05 ± 0.00^cA^
WA	5788 ± 83.91^aA^	24.33 ± 1.53^aB^	150 ± 4.58^bB^	4.84 ± 0.04^abA^	2.71 ± 0.03^aC^	3.21 ± 0.04^bB^	0.02 ± 0.00^cB^	0.07 ± 0.00^aA^	0.10 ± 0.01^aA^
WT	5883.67 ± 56.07^aA^	23 ± 1.00^abB^	162 ± 2.65^aB^	4.77 ± 0.08^bA^	2.77 ± 0.13^aC^	3.26 ± 0.05^bB^	0.03 ± 0.00^bB^	0.08 ± 0.02^aA^	0.08 ± 0.01^bA^
WE	5884 ± 12.77^aA^	23 ± 2.65^abB^	153.33 ± 2.89^bB^	4.92 ± 0.05^aA^	2.44 ± 0.45^aC^	3.24 ± 0.03^bB^	0.02 ± 0.00^cB^	0.14 ± 0.08^aA^	0.09 ± 0.00^abA^

Lowercase letters represent the differences in Bacteria, Fungi and Viruses of FS, WA, WT, and WE. Capital letters indicate differences in bacteria, fungi, and viruses among the FS, WA, WT, and WE groups.

Bacteria at phylum, genus and species level, as well as fungi and viruses at genus level were analyzed based on species annotation results. At the phylum level of bacteria (Figure 1B), microbiota was mainly composed of Firmicutes, Bacteroidetes, Proteobacteria, and Actinobacteria, among which Firmicutes and Bacteroidetes accounted for more than 90% of them (23). Bacteroidetes was a Gram-negative bacterial that plays an essential role in carbohydrate metabolism (24). The abundance of Bacteroidetes in WT was the highest among the three intervention groups and the lowest in WE group. Firmicutes were considered the major butyrate producers and major degraders of indigestible polysaccharides in the gut. The abundance of Firmicutes was highest in WE, and higher in WA than in WT. The ratio between these two phylum (the Firmicutes/Bacteroidetes (F/B) ratio) had been associated with maintaining homeostasis, and changes in this ratio could lead to various pathologies. Other studies have shown that a lower F/B ratio reduces obesity and intestinal inflammation (25, 26). WT had the lowest abundance of Firmicutes and the highest abundance of Bacteroidetes. Thus, the ratio of F/B was meager. The high starch and dietary fiber content of WT may also be responsible for the low ratio of F/B in the gut (27).

At the genus level in Figure 1C, *Bacteroides* were most abundant in FS and decreased in WT, WA, and WE. After *in vitro* fermentation, a large number of SCFAs were produced and the pH value declined, which inhibited the production of *Bacteroides* and thus reduced the abundance (28). *Lactobacillus* predominated in WA, but also took large proportions in WT and WE. *Lactobacillus* were facultatively anaerobic (29), which utilized carbohydrates fermentatively and produced lactate as a major end-product (30). Then *Bifidobacterium* predominated in WA, WT, and WE. *Bifidobacterium* were high G + C Gram-positive bacteria. *Bifidobacterium* was constantly used as the probiotic ingredient in many functional foods (31). Dietary fiber can promote the massive growth of *Bifidobacterium* and *Lactobacillus* (32). Consequently, the intervention of fiber-rich cereals mixture encouraged the development of both types of bacteria on a large scale. *Phocaeicola* was the most abundant in WT. It can help the host to establish a stable intestinal flora relationship and affect the health of the host (33), such as alleviating diseases, inhibiting the colonization of pathogenic bacteria and enhancing immunity (34). *Butyricicoccus* was more abundant in the three intervention groups and produced more butyrate. Butyrate had several beneficial properties that were essential to maintaining gastrointestinal health. Therefore butyrate-producing bacteria (*Butyricicoccus*) were seen as the next generation of probiotics (35). Furthermore, the effect of cereals mixture on bacterial abundance at the species level (Figure 1D). *Escherichia_coli* was a rod-shaped bacterium in *Enterobacteriaceae* (36). The bacteria mainly inhabited the intestinal tract of animals, including humans, which was conditional pathogenic bacteria (37). *Escherichia_coli* was significantly reduced in abundance by the three intervention groups and the abundance of this bacteria was extremely inferior in WE. *Faecalibacterium_prausnitzii*, anaerobic bacteria, was one of the main components of gut microbiota and the most essential butyrate-producing bacteria in the human colon (38). It was a promising anti-inflammatory bacterium that colonizes in the gut. Currently, this commensal bacterium has been considered as a bioindicator of intestinal health. The abundance of *Faecalibacterium_prausnitzii* significantly increased in the three intervention groups of WA, WT, and WE, with the highest abundance after WE treatment.

The cereals mixture also had a significant effect on the fungal community. There were strikingly differences in fungal community composition between FS and the three intervention groups. The fungal community composition of WA, WT, and WE all clustered together, but with subtle differences (Supplementary Figure 1A). To further analyze the differences in fungal abundance between the intervention groups, a sample-to-species relationship (Circos) analysis was performed at the genus level (Figure 1E). *Komagataella* was the genus in Ascomycota, which was a non-pathogenic, obligate aerobic budding yeast, which was beneficial to the human body, such as anti-depression and inhibiting inflammation, etc. (39). *Komagataella* was enriched in WE and also highly abundant in WA and WT. It showed that sweet buckwheat has a promoting effect on *Komagataella*. *Trichophyton* is a fungus that causes skin diseases in human and animals (40). It was most abundant in FS, significantly decreased in abundance after the three intervention groups, and was the lowest in WT. It indicated that after the intervention of the three groups, the harmful fungi were inhibited, and the inhibition effect of tartary buckwheat was the most obvious.

PCoA analysis of viruses was performed (Supplementary Figure 1B), and the result showed that the FS community differed considerably from the three intervention groups. The distribution of viruses species in WT was significantly different from that in WA and WE. WA and WE were more similar in viral composition. Furthermore, we analyzed the viruses abundance of each group at the genus level (Figure 1F). *Taranisvirus* and *Eponavirus* predominated in WA, WT, and WE. The abundance of *Oslovirus* decreased significantly in the intervention group. Taken together, sweet and tartary buckwheat could alter the structure of gut microbiota.

### Analysis of SCFAs and lactate in the gut

**FIGURE 2:**
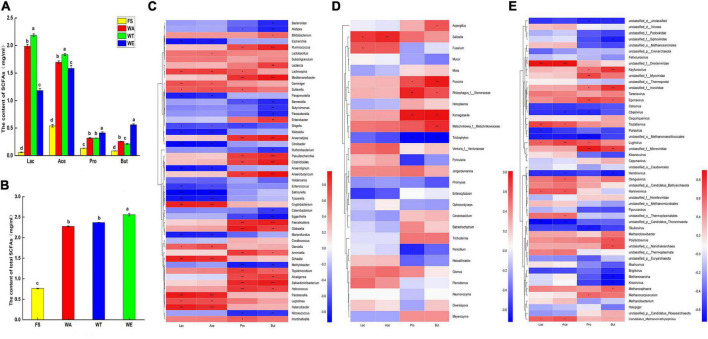
The content of acetate, propionate, butyrate and lactate in different samples after fermentation and Spearman correlation heat map of species and metabolites in cereals mixture; **(A)** Four organic acids in each group content; **(B)** Total content of SCFAs. Letters represent the significance (*P* < 0.05) of single metabolite differences in different samples. **(C)** Correlation of bacteria at genus level with lactate, acetate, propionate, butyrate; **(D)** Correlation of fungi at genus level with lactate, acetate, propionate, butyrate; **(E)** Correlation of virus at genus level with lactate, acetate, propionate, butyrate; color intensity is proportional to the relative abundance of the genus. Correlation coefficients are represented by colors. Dark red indicates positive correlation and dark blue indicates negative correlation. *P* values calculated using Spearman’s rank correlation test, *P < 0.05; ^**^P < 0.01; ^***^P < 0.001.

SCFAs were generally considered beneficial to human health. It was the main product of microbial carbohydrate fermentation (41), which could inhibit the growth of pathogenic bacteria in the intestine by changing the intestinal pH, and it had the function of regulating the immune system and promoting lipid metabolism (42). In this study, the concentrations of lactate, acetate, propionate and butyrate were analyzed after fermentation of the cereals-feces mixture (Figure 2A). The formic acid in SCFAs was not detected, probably because their concentrations were too little to be detected. Among the total SCFAs, the content of acetate was the highest, followed by propionate and butyrate. Acetate was the primary source of bacteria to provide energy to the host, and it also inhibits colitis. Results of others showed that diets deficient or low in dietary fiber exacerbate colitis development, while greatly high intake of dietary fiber or the acetate protects against colitis (43). Butyrate was a major energy source for colonic epithelial cells, reduced intestinal mucosal permeability, promoted intestinal barrier restoration, and prevented or reduced the incidence of colon cancer (44), Butyrate may inhibit the growth of cancerous colonocytes, and energy homeostasis necessary for the rapid cell proliferation of the colonic epithelium (45, 46), and propionate had positive effects on metabolic health, such as lowering serum cholesterol (47). The total content of SCFAs in both WE and WT were very high, which indicated that sweet and tartary buckwheat had completely significant effects on the intestinal health. Several studies had shown that resistant starch and non-starch polysaccharides, when present in the cereals mixture, were often associated with the high production of SCFAs by the human gut microbiota (48). This might be the reason for the high levels of total SCFAs in the three intervention groups. The amount and type of SCFAs in the gut could be affected by the microbiota composition, the availability of dietary fiber and its physicochemical properties. The starch content and dietary fiber content of WT and WE were higher than that of WA. Therefore, sweet and tartary buckwheat produced more SCFAs than wheat.

Bacteroidetes were most abundant in WT. It produced a large number of carbohydrate-degrading enzymes, and it could degrade carbohydrates to produce pyruvate, which produced large amounts of lactate and acetate (49). Lactate and acetate were most abundant in WT (Figure 2C). In general, tartary buckwheat produced a lot of lactate and acetate under the promotion of Bacteroidetes. *Ruminococcus* also produced carbohydrate-degrading enzymes and thus could ferment carbohydrates to produce SCFAs such as propionate and butyrate. The contents of propionate and butyrate in WA and WT were similar and lower than in WE. *Ruminococcus* was enriched in WE, which may account for the abundance of propionate and butyrate in WE. *Faecalibacterium* mainly produces butyrate, which has a positive effect on intestinal tract and metabolism. And it also exerted protective effects against colon, which ameliorated gut dysbiosis, with an increase in bacterial diversity and the abundance of SCFAs (50). *Faecalibacterium* was dominant in WE, This bacteria might have resulted in WE having the highest butyrate content and the highest total acid content. And it could be considered that sweet buckwheat has an inhibitory effect on colitis. *Bifidobacterium* could produce large amounts of lactate and acetate in carbon-rich environments. And *Bifidobacterium* was enriched in WA. Thus, WA also produced a lot of lactate and acetate.

In short, the content of total acid (Figure 2B) showed that the ranking of total acid content in the sample group was WE, WT and WA. There were significant differences in entire acid content between the three intervention groups and the FS group. Therefore, the results showed that the three intervention groups still had a great impact on the production of intestinal flora metabolites.

### Correlation analysis between gut microbiota with lactate and SCFAs

LEfSe identified significant bacteria at the genus level of FS, WA, WT, and WE, for correlation analysis (Supplementary Figure 2). And correlations of bacteria and SCFAs were analyzed (Figure 2C). The abundance of *Cryptobacterium, Schaalia, Lachnospira* and other genera in WT was higher than that in WA and WE. *Cryptobacterium, Schaalia, Lachnospira* and *Lactobacillus* were positively correlated with lactate and acetate. Among them, fructose bisphosphate aldolase (EC 4.1.2.13) has high activity in the cell extract of *Lachnospira*. It was a key enzyme in the glycolysis and pentose phosphate pathways (51). *Paraprevotella, Klebsiella, Anaerotignum, Salmonella*, and *Tyzzerella* were significantly negatively correlated with lactate and acetate. In Glycolysis/Gluconeogenesis metabolism, Starch produced phosphoenol-pyruvate through EC 4.1.2.13, and produced pyruvate through pyruvate kinase (EC 2.7.1.40) and entered pyruvate metabolism, involving 2-oxoacid oxidoreductase (EC 1.2.7.11), pyruvate oxidoreductase (EC 1.2.7.1) and L-lactate dehydrogenase (EC 1.1.1.27) generate lactate and acetate.

The genera *Ruminococcus, Bifidobacterium, Enterobacter, Anaerostipes*, and *Gibbsiella* were positively correlated with propionate and butyrate. *Methylobacter* and *Nitrosococcus* were negatively correlated with propionate. *Bacteroides, Alistipes*, and *Eggerthella* were significantly negatively correlated with butyrate. *Ruminococcus* produced pullulanases, glycoside hydrolase family 13 (GH13) enzymes, which were carbohydrate-degrading enzymes that ultimately produced propionate and butyrate (52). *Enterobacter*, one of the major butyrate-producing genera, was most abundant in WE. It was positively correlated with butyrate, but negatively correlated with lactate and acetate. This might be the reason why butyrate was abundant in WE, while lactate and acetate were low. *Anaerostipes* were considered a key gut microbe associated with human health and disease, and they were capable of producing butyrate (53). *Saitoella* was positively correlated with lactate and acetate. *Komagataella* and propionate, butyrate was positively correlated. Also, viruses such as *Eponavirus, Taranisvirus* and lactate, acetate, propionate and butyrate were positively correlated (Figures 2D,E). The results showed that although fungi and viruses make up a small proportion of the healthy human gut, they also have important effects on the production of metabolites.

### Functional gene and metabolic pathway analysis

**FIGURE 3:**
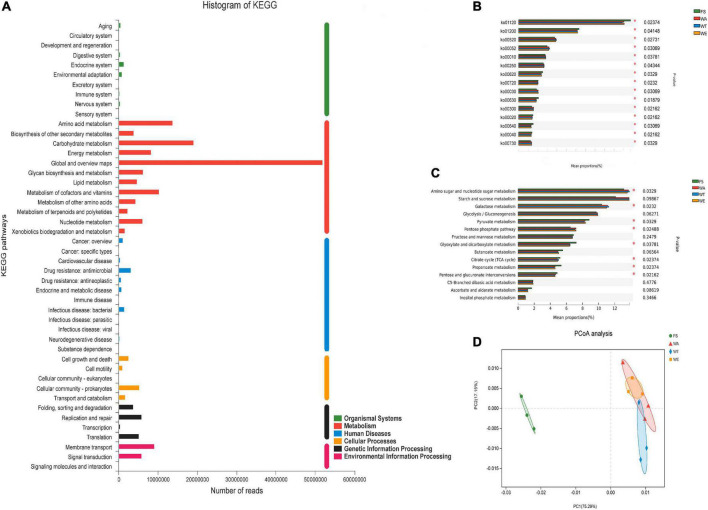
Composition of the KEGG system and analysis of differences between groups. **(A)** Distribution of annotated genes of cereals mixture samples in KEGG; **(B)** Comparison of multiple groups of metabolism pathway at the second level of KEGG; **(C)** Comparison of multiple groups of carbohydrate metabolism pathway at the third level of KEGG; **(D)** Principal coordinate analysis (PCoA) of KEGG.

The sequenced genes were annotated into six modules of the KEGG pathway, including: Metabolism, Genetic Information Processing, Environmental Information Processing, Cellular Processes, Organic Systems, and Human Diseases (Figure 3A). According to the KEGG annotation results, the differential in the two-level metabolic pathways among the groups were analyzed (Figure 3B). There were five metabolic pathways, including ko00520 (Amino sugar and nucleotide sugar metabolism), ko00052 (Galactose metabolism), ko00010 (Glycolysis/Gluconogenesis), ko00620 (Pyruvate Metabolism) and ko00250 (Alanine, aspartate and glutamate metabolism), were highly abundant and dominant status. To further investigate the functional differences of KEGG among the samples of each group, the differentially expressed ko was annotated to the tertiary classification level (Figure 3C). The results showed that Amino sugar and nucleotide sugar metabolism, Galactose metabolism, Pyruvate metabolism, Pentose phosphate pathway, Glyoxylate and Dicarboxylate metabolism, Citrate cycle (TCA cycle), Pentose and glucuronate interconversions and Propanoate metabolism were significantly different among different groups. Based on the differences analysis showed Amino sugar and nucleotide sugar metabolism, Galactose metabolism, Citrate cycle (TCA cycle) and Glyoxylate and dicarboxylate metabolism were the most abundant in WT. Galactose metabolism produces Galactose, which is crucial for early human development, with an established role in energy delivery and galactosylation of complex molecules (54). TCA cycle is a central route for oxidative phosphorylation in cells, and fulfills their bioenergetic, biosynthetic, and redox balance requirements (55). Pyruvate metabolism, Pentose phosphate pathway, Butanoate metabolism and Propanoate metabolism were abundant in WE. Pyruvate is the end-product of glycolysis, is derived from additional sources in the cellular cytoplasm, and is ultimately destined for transport into mitochondria as a master fuel input undergirding citric acid cycle carbon flux (56). Butanoate metabolism mainly produces butyrate, which was also the reason for the high content of butyrate in WE. PCoA analysis showed that there were significant differences between the three intervention groups and FS. (Figure 3D). The results showed that cereals mixture increased the abundance of functional genes in metabolic pathways, which in turn better influenced the expression of key genes in the human gut microbes.

**FIGURE 4:**
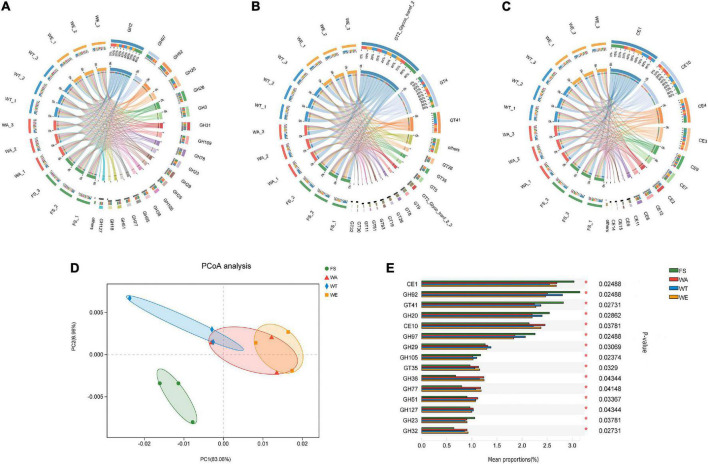
Composition and variance analysis of CAZymes groups. **(A)** Distribution and relative abundance of GH family; **(B)** distribution and relative abundance of GT family; **(C)** distribution and relative abundance of CE family; **(D)** CAZymes principal coordinate analysis (PCoA); **(E)** CAZymes multi-group comparison, the test method is “Tukey-Kramer”, the significance level is “0.99”, *P < 0.05; ^**^P < 0.01; ^***^P < 0.001.

The CAZy database focused on carbohydrate-active enzymes that degrade, modify or generate glycosidic bonds, and was a professional database for the study of related enzymes, including Glycoside Hydrolases (GHs), Glycosyl Transferases (GTs), Polysaccharide Lyases (PLs), Carbohydrate Esterases (CEs), Auxiliary Activities (AAs) and Carbohydrate-Binding Modules (CBMs). The results showed that the detected functional gene were enriched into 59 CBM families, 15 CE families, 230 GH families, and 68 GT families. GHs were a class of enzymes that bond between carbohydrate molecules. GH2 was the most abundant enzyme in the GHs family. They were followed by GH97, GH92, GH20, GH28, GH3, GH31, GH109, GH78, GH23, GH29, GH25, and other 10 families with higher abundance in the GH family (Figure 4A). Abundant GH2 (Glycosidase) acts as auxiliary enzymes to complete the digestion of plant polysaccharides and may also be involved in the degradation of carbohydrates derived from microorganisms in feces. GH20 (β-N-acetyl-d-hexosaminidase) catalyzes the hydrolysis of glycosidic bonds in sugars, glycoproteins and glycolipids. Defects in human GH20 lead to lysosomal storage diseases, Alzheimer’s disease, and osteoarthritis (57). α-l-fucosidases were most abundant in GH29 (Fucosidase), and transfer glycosylation catalyzed by retention of α-l-fucosidases was a novel pathway for the manufacture of biomimetic HMOs (Human Milk Oligosaccharides). HMOs constitute a unique family of bioactive lactose molecules present in human milk. HMOs were critical for infant health and development (58), so the highest production of GH29 in the WT group was beneficial. CEs were enzymes that catalyze the esterification of sugars (59). There were 15 CE families in total (Figure 4B), among which CE1, CE10, CE3, CE9, and CE7 had high abundance. Then CE1, CE3, and CE7 are xylan esterases acting on xylan branches (60). CE9 was a phosphate deacetylase. GTs were present in tissues such as kidney, pancreas and liver. A total of 68 GT families were annotated in this study. Several highly abundant families were GT2_Glycos_transf_2, GT4, GT41, GT28, GT35, and GT5 (Figure 4C). GT was related to the biosynthesis of oligosaccharides and polysaccharides (61), and the higher relative abundance of GT41 represents the presence of polysaccharide-producing microorganisms in the samples. Polysaccharides had inoxidizability, immunomodulatory, tumor suppressor and gut microbiome modulating effects. PCoA analysis showed that the WA, WT, and WE were mainly focused on one quadrant (Figure 4D). Among the 15 enzyme families in Figure 4E, the abundance of CE10, GH29, GT35, GH36, GH77, GH51, GH127, and GH32 in the FS group was lower than that in the WA, WT, and WE. Most of the enzymes were significantly changed after treatment in the intervention group, and this might be due to the decrease in pH resulting from the production of large amounts of SCFAs, which affects CAZymes gene expression and carbohydrate substrate consumption.

### Species and functional contribution analysis

**FIGURE 5:**
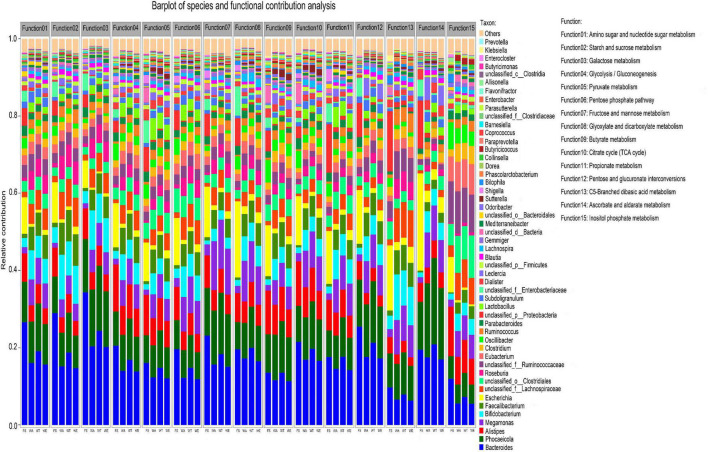
Relative contributions of different taxa to metabolic pathways at the genus level. Function1 to Function15 are the functional name of the level 3 KEGG pathway, and the ordinate is the corresponding genus-level contributing microorganism.

The overall functional characteristics of 50 strains with high abundance were detected. According to the KEGG annotation results showed that the metabolic capabilities of 50 strains in 15 KEGG functions were different (Figure 5). The results showed that *Bacteroides, Phocaeicola, Alistipes, Bifidobacterium* and *Faecalibacterium* in the four groups were all major contributors to the 15 KEGG functions.

*Bacteroidetes* in WT contributed the most among several functions of Glyoxylate and dicarboxylate metabolism, Propionate metabolism, Ascorbate and Aldarate metabolism. The contribution of *Lactobacillus* to all functions was also prominent, especially in the metabolic pathways of the intervention groups. *Lactobacillus*, the key genus of lactate bacteria group, plays functional roles in the human body (62, 63). Several *Lactobacillus* species were often used as probiotics and could benefit host health when administered in adequate amounts (64, 65). *Prevotella* contributed to multiple functionality. It promoted an increase in glycogen storage and played an important role in sugar metabolism (66). *Prevotella* was also one of the important bacteria producing propionate (67). Many genera in WA, WT and WE such as *Sutterella* and *Lactobacillus* had a high contribution to Function 5 (Pyruvate metabolism), suggesting that pyruvate production in cereals mixture was involved in these genera. Pyruvate was the end-product of glycolysis. EC 2.7.1.40 (pyruvate kinase) and EC 1.1.1.27 (lactate dehydrogenase) were key enzymes that produce pyruvate. EC 2.7.1.40 catalyzes the dephosphorylation of phosphoenolpyruvate into pyruvate in glycolysis. In the three intervention groups, *Eubacterium* and *Faecalibacterium* contributed significantly to Function 9 (butyrate metabolism). They were the major butyrate-producing genera. The final steps in butyrate synthesis by *Faecalibacterium* could occur via EC 2.7.2.7 (butyrate kinase) and phosphotransbutyrylase or via butyryl-coenzyme A (CoA): acetate CoA-transferase (EC 2.8.3.18) with net consumption of acetate (68). EC 2.7.2.7 was predominant in WT and also very abundant in WA and WE. But the butyrate concentration was highest in WE but not in WT, so EC 2.7.2.7 may not be the predominant butyrate-producing enzyme in WE. However, the abundance of EC 2.8.3.18 in WE was significantly higher than that in the other intervention groups. Therefore, EC 2.8.3.18 was the main butyrate-producing enzyme in cereals mixture. The genera that contributed significantly to Function 11 (Propionate metabolism) were *Ruminococcus* and *Prevotella* in the three intervention groups. Two pathways were known for the propionate formation from fermentation by gut bacteria. *Ruminococcus* and *Prevotella* were two of these genera. Most hexose and pentose sugars were processed through the succinate pathway. Whereas the deoxy sugars fucose and rhamnose were metabolized by the propanediol pathway (69). The succinate pathway was found mainly in Bacteroidetes and Firmicutes. It was the key route for propionate formation from carbohydrates driven by the abundant Bacteroidetes. Therefore, these bacteria in the human gut could regulate various metabolic pathways, which in turn producing different metabolites.

**FIGURE 6:**
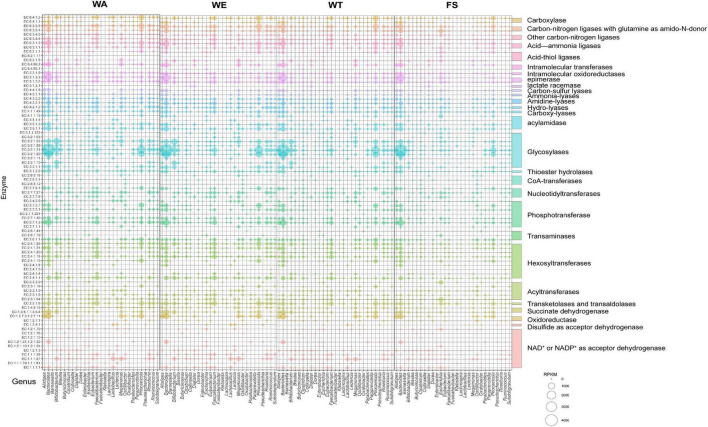
Microbial taxonomic distribution and enzyme gene abundance of substrate decomposition and metabolite synthesis genes during the fermentation of cereals mixture. The RPKM value of the enzyme encoding gene was proportional to the diameter of the bubble. The vertical coordinate represents the EC number of the functional enzyme. The horizontal axis shows only 29 key genera with relative contributions of functional genes.

The contribution of different taxa was further assessed by calculating the abundance of key enzyme genes and biological taxa in each set of samples. According to the CAZy annotation results, a total of 83 enzymes were identified, and these enzymes were divided into 29 families (Figure 6). Among these enzyme families, Glycosylases, Phosphotransferase, Hexosyltransferases, Acyltransferases, NAD + or NADP + as acceptor dehydrogenase were more abundant. EC 2.7.1.2 (glucokinase) was significantly expressed in the three intervention groups by *Bacteroides, Alistipes, Faecalibacterium, Parabacteroides*, and *Roseburia*, through which α-D-Glucose catalyzed the production of pyruvate through a series of metabolic pathways, which in turn produced lactate and SCFAs. EC 1.1.1.27 was significantly associated with *Bifidobacterium, Lactobacillus* and *Megamonas* in the WA and WT, producing lactate via ko00620 (Pyruvate metabolism). *Bacteroides, Alistipes, Megamonas*, and *Phocaeicola* with EC 1.2.7.11(2-oxoacid ferredoxin oxidoreductase subunit alpha) were significantly correlated in WA and WT. EC 1.2.7.11 catalyzes acetyl-CoA to acetate. EC 6.2.1.1 was the key enzyme that catalyzes the production of propionate from propionyl-CoA, and it was significantly related to *Bacteroides* and *Phocaeicola* in WE. *Alistipes, Bacteroides*, and *Phocaeicola* in WE were significantly expressed with EC 2.7.2.7, producing butyrate via the butyrate metabolic pathway. The results showed that *Alistipes, Bacteroides, Bifidobacterium, Phocaeicola* had the strong metabolic ability and a more outstanding contribution to the expression of enzymes. Under the action of the above microorganisms, macromolecular carbohydrates were degraded, providing beneficial conditions for the growth of various microorganisms.

### Metabolic pathways of SCFAs produced after *in vitro* fermentation

**FIGURE 7:**
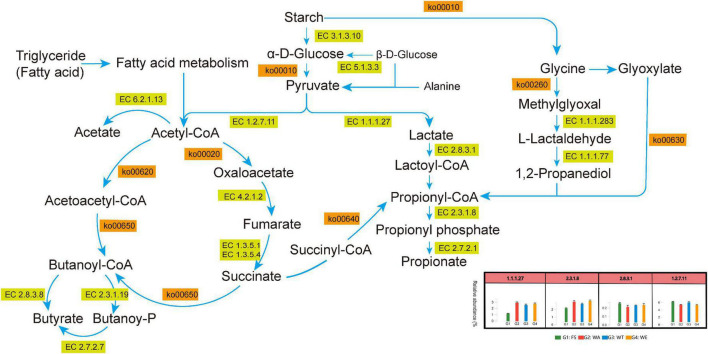
Metabolic pathways of SCFAs produced after *in vitro* fermentation.

Several metabolic pathways with high abundance could be shown in Figure 3B. At the same time, the metabolic pathways of pyruvate to lactate, acetate, propionate and butyrate through different metabolic transformations were also constructed. (Figure 7). Combined with the KEGG metabolic pathway annotation, in addition to several highly abundant metabolic pathways in Figure 3B, there were several key metabolic pathways: ko00010 (Glycolysis/Gluconogenesis), ko00640 (Propionate Metabolism), ko00260 (Glycine, serine and threonine metabolism), ko00630 (Glyoxylate and Dicarboxylate Metabolism), ko00020 (Citrate cycle ((TCA) cycle)), ko00720 (Carbon Fixation Pathways in Prokaryotes), ko00650 (Butyrate Metabolism), ko00620 (Pyruvate Metabolism, etc. The main related substances were: pyruvate, lactate, lactoyl-CoA, propionyl-CoA, propionate, acetyl-CoA, acetate, butanoyl-CoA, butyrate, etc. In ko00520 (metabolism of Amino sugar and nucleotide sugar), Chitosan produced GlcNAc via EC 3.2.1.52 (beta-N-acetylhexosaminidase), and then catalyzed Fru-6P via EC 3.5.99.6 (glucosamine-6-phosphate deaminase). Finally, it entered ko00010 (Glycolysis/Gluconogenesis) to produce lactate and acetate. These two enzymes were most abundant in WT, the reason that accounts for the high abundance of lactate and acetate in WT. ko00250 (Alanine, aspartate and glutamate metabolism) produced 2-Oxoglutarate through EC 3.5.1.3 (omega-amidase), and then entered the ko00650 pathway to produce butyrate. (S)-Malate catalyzes the generation of fumarate through EC 4.2.1.2 (fumarate hydratase, class I) in ko00620 (Pyruvate metabolism), and produced succinate in EC 1.3.5.4 (fumarate reductase flavoprotein subunit) and enters ko00640 (Propionate metabolism) produced propionate. EC 4.2.1.2 and EC 1.3.5.4 were abundant in WE. This might be the reason why WE produced more propionate.

In the ko00010 pathway, pyruvate could form lactate under the catalysis of EC 1.1.1.27. In the ko00640 pathway, EC 2.8.3.1 catalyzes lactate to lactoyl-CoA, and then lactoyl-CoA to propionyl-CoA, and through EC 2.7.2.1 catalytically generate propionate. EC 2.8.3.1 was most abundant in WE. These two enzymes play an important role in the production of propionate, so the content of propionate in WE was notably high (Figure 2A). Although succinyl-CoA also generates propionyl-CoA, there were few intermediate-catalyzed enzymes, so the generated propionate was also extremely few. L-Lactaldehyde produced by Glycine in the ko00260 pathway could also be converted to lactoyl-CoA. Glyoxylate also produced propionyl-CoA in ko00630. Pyruvate in the ko00010 pathway could generate acetyl-CoA, which was catalyzed to acetate by EC 6.2.1.13. The reason for the high concentration of acetate was that pyruvate was catalyzed by the highly abundant EC 1.2.7.11 to acetyl-CoA, which in turn generates acetate. The highest acetate in WT was also because the abundance of EC 1.2.7.11 in WT was higher than that in WA and WE. acetyl-CoA generates Oxaloacetate in ko00020, and then catalyzes a series to generate succinate, which generates butanoyl-CoA through ko00650 pathway, and finally catalyzes to generate butyrate by EC 2.8.3.8. The participation of different intervention groups affected the expression of genes encoding enzymes, which affected the production of SCFAs, thus affecting intestinal health.

## Conclusion

Sweet and tartary buckwheat had regulatory effects on gut microbial composition and metabolic pathways. The intestinal beneficial flora in WT and WE increased significantly after treatment with sweet and tartary buckwheat, such as *Faecalibacterium* and *Ruminococcus*. The contents of starch and dietary fiber were higher in WT and WE than in WA. A large amount of dietary fiber could change the composition and metabolism of intestinal flora and promote intestinal health. And starch could produce sugars through a series of metabolic pathways, then produced SCFAs. The total content of SCFAs in the three intervention groups was also significantly affected by the gut microbiota after cereals mixture treatment, with more lactate and acetate produced in WT and more propionate and butyrate produced in WE. According to the annotation results of KEGG and CAZy, lactate, acetate, propionate and butyrate were produced through pyruvate, acetate and butyrate metabolism etc. The key enzymes involved are EC 1.1.1.27 (lactic dehydrogenase) and EC 1.2.7.11(2-oxoacid ferredoxin oxidoreductase subunit alpha). Therefore, it indicated that sweet and tartary buckwheat had certain beneficial regulation on the human intestinal flora, functional genes and metabolites SCFAs. The research results could provide reference and some theoretical basis for sweet and tartary buckwheat to improve intestinal flora and promote intestinal health.

## Data availability statement

The data presented in this study are deposited in the NCBI repository, accession number: SRP372133.

## Ethics statement

The studies involving human participants were reviewed and approved by Science and Technology Ethics Committee of Heilongjiang Bayi Agricultural University. The patients/participants provided their written informed consent to participate in this study.

## Author contributions

DY, DZ, and QY contributed to the conception and design of the study. ZL and CW organized the database. TS, LM, XW, MW, and LX performed the statistical analysis. DY and QY wrote the first draft of the manuscript. All authors contributed to the manuscript revision, read, and approved the submitted version.

## Footnotes

1
www.illumina.com

2
https://github.com/OpenGene/fastp

3
http://cloud.majorbio.com/

4
https://github.com/bbuchfink/diamond

5
https://www.kegg.jp/

6
http://bcb.unl.edu/dbCAN2/download/Databases/

7
https://cloud.majorbio.com/
