# Supplementary material for: Wheat supplement with buckwheat affect gut microbiome composition and circulate short-chain fatty acids
Source: Front Nutr. 2022 Sep 6;9:952738. doi: 10.3389/fnut.2022.952738 (PMC9486400; doi:10.3389/fnut.2022.952738)
Supplement: Supplementary file 1 [file Data_Sheet_1.pdf]

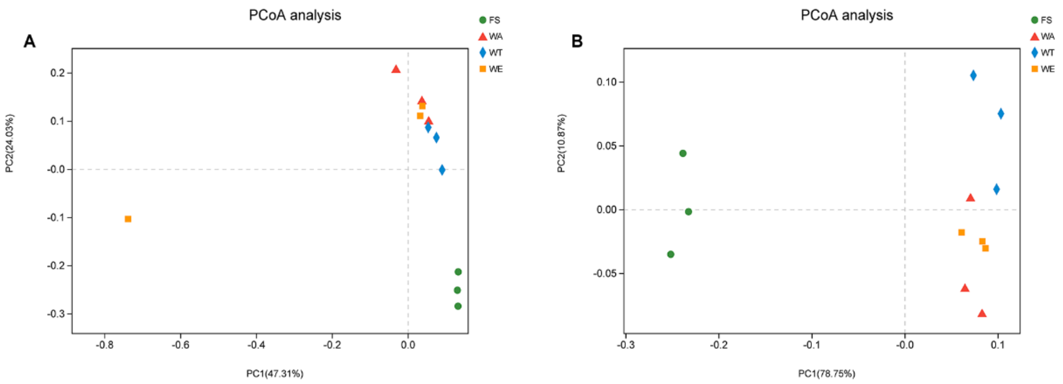

Figure S1. Principal coordinate analysis (PCoA) of fungi and viruses, (genus level). (**A**) Fungal principal coordinate analysis; (**B**) Viruses principal coordinate analysis.

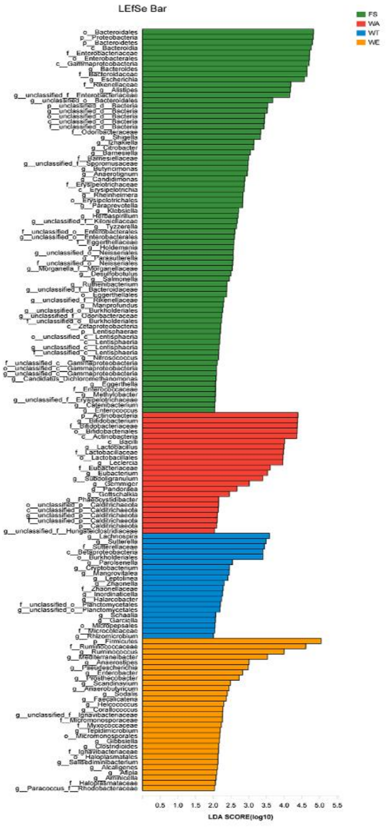

Figure S2. Linear discriminant analysis (LDA) of bacteria with a threshold of 2.
